# Ultrasound stimulation of the vagal nerve improves acute septic encephalopathy in mice

**DOI:** 10.3389/fnins.2023.1211608

**Published:** 2023-07-17

**Authors:** Yukio Imamura, Hisatake Matsumoto, Jun Imamura, Naoya Matsumoto, Kazuma Yamakawa, Nao Yoshikawa, Yuki Murakami, Satoko Mitani, Junichiro Nakagawa, Tomoki Yamada, Hiroshi Ogura, Jun Oda, Takeshi Shimazu

**Affiliations:** ^1^Department of Traumatology and Acute Critical Medicine, Graduate School of Medicine, Osaka University, Osaka, Japan; ^2^Organization for Research Initiatives and Development, Doshisha University, Kyoto, Japan; ^3^Department of Architectural and Environmental Planning, Graduate School of Engineering, Kyoto University, Kyoto, Japan; ^4^Department of Hygiene and Public Health, Kansai Medical University, Osaka, Japan; ^5^Molex Corporation, Ltd., Yamato, Kanagawa, Japan; ^6^Department of Emergency Medicine, Osaka Medical and Pharmaceutical University, Osaka, Japan; ^7^Human Health Science, Graduate School of Medicine, Kyoto University, Kyoto, Japan; ^8^Faculty of Acupuncture and Moxibustion, Meiji University of Integrative Medicine, Kyoto, Japan

**Keywords:** septic encephalopathy, vagal nerve, ultrasound, α7 nicotinic acetyl choline receptor, proteomics

## Abstract

Septic encephalopathy (SE) is characterized by symptoms such as coma, delirium, and cognitive dysfunction, and effective therapeutic interventions for SE remain elusive. In this study, we aimed to investigate the potential alleviating effects of vagal nerve stimulation (VNS) on SE-associated signs. To evaluate our hypothesis, we utilized a mouse model of SE induced by intraperitoneal injection of lipopolysaccharide (0.3 mg per mouse) and administered noninvasive, high-frequency ultrasound VNS. To assess the efficacy of ultrasound VNS, we measured inflammation-related molecules, including the α7 nicotinic acetylcholine receptor (α7nAChR) expression in peritoneal macrophages and plasma interleukin 1β (IL-1β) levels. Consistent with our hypothesis, SE mice exhibited reduced α7nAChR expression in macrophages and elevated IL-1β levels in the blood. Remarkably, VNS in SE mice restored α7nAChR expression and IL-1β levels to those observed in control mice. Furthermore, we evaluated the effects of VNS on survival rate, body temperature, and locomotor activity. SE mice subjected to VNS demonstrated a modest, yet significant, improvement in survival rate, recovery from hypothermia, and increased locomotor activity. To investigate the impact on the brain, we examined the hippocampus of SE mice. In control mice, VNS increased the expression of c-fos, a marker of neuronal electrical excitability, in the hippocampus. In SE mice, VNS led to the restoration of aberrant firing patterns in hippocampal neurons. Additionally, proteomic analysis of hippocampal tissue in SE mice revealed abnormal increases in two proteins, tissue factor (TF) and acyl-CoA dehydrogenase family member 9 (ACAD9), which returned to control levels following VNS. Collectively, our findings support the value of exploring the beneficial effects of ultrasound VNS on SE.

## Introduction

Sepsis is a life-threatening condition with an uncontrolled and abnormal immune response to overwhelming infection (Nedeva, 2021). After the microbial and viral infection, the immune system overreacts to the infection, leading to multiple organ dysfunctions (Angus and van der Poll, 2013). Notably, septic encephalopathy (SE) occurs secondary to infection without overwhelming central nervous system infection (Gofton and Young, 2012). Moreover, from a clinical perspective, SE patients usually suffer from coma, delirium, and cognitive dysfunction (Gofton and Young, 2012). Several lines of evidence suggested that SE occurred because of inflammatory cell activation, upregulated cytokines, blood–brain barrier (BBB) disruption (Imamura et al., 2011), acetylcholine hypofunction (Zhang et al., 2014), and neuronal cell death (Iacobone et al., 2009). To date, the pathophysiology and underlying molecular mechanisms causing SE are only incompletely understood (Chung et al., 2020). In clinical routine, there is no specific recommendation for a standardized pharmacological treatment (Chung et al., 2020). Recently, preclinical trials that have been established to treat and mitigate SE consequences using mesenchymal stem cell therapy (Lima et al., 2022) were challenged. However, the efficacy of the treatment is limited, and it is necessary to further explore effective therapeutic options. Since no therapeutic method has been established clinically at this time, we hypothesized that ultrasound stimulation would be effective, and we demonstrated its effectiveness (whether or not and the rate of recovery) by testing this hypothesis.

The vagus nerve (VN)—part of the cholinergic anti-inflammatory system—regulates the innate inflammatory response (Tracey, 2009). When VN is electrically stimulated, the efferent pathway of VN induces the release of acetylcholine, which interacts with the α7 nicotinic acetylcholine receptor (α7nAChR), thereby reducing the inflammatory responses (Floto and Smith, 2003). α7nAChRs are expressed on the cell membrane of lymphocytes, including macrophages, and their activation inhibits cytokine production (Rosas-Ballina et al., 2011).

Several lines of evidence indicate that the activation of the cholinergic anti-inflammatory pathway improves the septic condition of mice (Wang et al., 2004; van Westerloo et al., 2005). Compared with other therapeutic interventions in an animal sepsis model, the activation of the cholinergic anti-inflammatory pathway remarkably improves the condition of the animals and immunocompromised patients after sepsis (Peña et al., 2011). In the animal sepsis model, the pathway was activated by directly applying electrical pulses to surgically isolated VN, which is difficult for SE patients. Therefore, developing a noninvasive method to stimulate VN is necessary for SE patients.

Ultrasound is sound waves with frequencies of >20,000 Hertz (Hz). Ultrasound imaging or sonography is widely used to visualize the inner organs for establishing a medical diagnosis. In contrast, ultrasonic waves can noninvasively pass into the organ without causing any damage. Evidence suggests that ultrasonic waves exert increased electrical excitability on neurons (Ye et al., 2018; Yu et al., 2021). In this study, we applied ultrasound to VN and explored the novel therapeutic potential of noninvasive ultrasound stimulation in SE. Accordingly, we propose noninvasive ultrasound VN stimulation (VNS) as a novel approach for achieving a better therapeutic effect in SE.

## Materials and methods

### Mice

The included mice were nonstarved males (age, 12 weeks) with C57/BL/6 J genetic background; they were purchased from Nihon SLC (Hamamatsu, Japan). The Animal Care and Use Committee of Doshisha University (A21011) and Osaka University Medical School (25–094-000) approved the surgical procedure. All experimental procedures were in accordance with ARRIVE guidelines. In total, 163 mice were used in this study.

### A mouse model of SE

Mice were deeply anesthetized using the intraperitoneal (i.p.) administration of medetomidine (0.3 mg/kg), midazolam (4.0 mg/kg), and butorphanol (5.0 mg/kg). Sepsis was induced by intraperitoneal injection of LPS (0.3 mg/mouse). Based on our previous paper examining functional brain changes in a mouse model of SE (Imamura et al., 2016), we confirmed whether SE was induced by sensory stimulation of the mice after SE induction and the blunting of the returning response. They were randomly divided into four groups: CON, LPS-administered, LPS + VNS3h (i.e., VNS at 3 h after LPS administration in mice), and LPS + VNS18h mice (i.e., VNS at 18 h or 36 h after LPS administration in mice). Sepsis was induced by a 0.3 mg/mL i.p. injection of LPS (LPS from *Escherichia coli* 055: B5, Sigma-Aldrich, St Louis, MO) according to the method described in our previous report (Imamura et al., 2011; Wang et al., 2013). Blood plasma samples were collected from all mice and tested using ELISA assay to determine whether SE had occurred. Saline was injected subcutaneously (s.c.) at a dose of 35 mL/kg to prevent dehydration. For achieving better recovery from SE, all groups were given free access to Diet Gel 93 M (ClearH_2_O, Portland, ME, United States) and water. Mice that died during experiment were excluded from the data (10 individuals were lost). For a line of experiments, two experimenters were participated to do the mice experiments blinded: experimenter 1 provided with blinded mouse to experimenter 2, and experimenter 2 performed the experiments. Then, experimenter 1 analyzed the data.

### Ultrasound stimulation

#### Characterization of ultrasonic waves and their effectiveness on VN excitability

The two ultrasound devices used in this study have different frequencies, i.e., 300 kHz (Murata Manufacturing Co. LTD., Kyoto, Japan) and 1.0 MHz (FUJI Ceramix, Japan). These devices were connected to a function generator (Instek Japan Corp, Kanagawa, Japan) and worked as an emitter. They were also connected to the amplifier and worked as a receiver. The intensity of the received ultrasonic wave was converted to electrical excitability. The voltage difference was recorded using a portable electric amplifier (EBA-100, Unique medical, Co. Osaka, Japan).

#### Therapeutic functional recovery from SE

We used commercial ultrasound therapy (US-731, ITO, Kawaguchi, Saitama, Japan). The apparatus was sequentially attached to the bilateral sides of the cervical VN of the mice. The stimulation was performed on the cervix of mice, with an intensity of 3.0 W/cm^2^ and duty of 50% for 20 min. During the ultrasonic stimulation, the mice were deeply anesthetized and maintained in an air-conditioned room (28°C).

#### Recording of evoked electrical excitability in VN

After deep anesthesia, an electric razor was used to shave the hair on the neck area of the mice. Before cutting the skin, it was sterilized using 70% ethanol. Using surgical scissors, the center line of the skin was cut vertically from the sternum to the mandible (approximately 2 cm long) and the thymus gland was gently teased out. For unilateral isolation, after carefully separating the fat and connective tissue, the right jugular VN was exposed from beneath the carotid artery. A glass electrode filled with 3 M KCl was then placed in the VN. The electrode was inserted and the action potentials in the VN were recorded. This recording was connected to an electrical amplifier (UAS-400, Unique medical, Co. Osaka, Japan). Evoked potentials in the VNS were then recorded.

#### Macrophage

The peritoneal macrophage was isolated using a previously described method (Zhang et al., 2008). Briefly, ice-cold phosphate-buffered saline (PBS) stored in a 10-mL syringe with a 23G needle was injected through the peritoneal wall along the left side of the mouse (splenic side). We injected 10 mL of PBS in each mouse. In the case of a punctured intestine, the mouse was removed and discarded to avoid contamination. Subsequently, the mice were shaken for 3 min to sufficiently suspend the macrophages in PBS. Using the same syringe and needle, PBS-containing macrophages were collected from the peritoneum. Typically, approximately 8 mL could be collected from each mouse. Thereafter, 2 mL of ice-cold red blood cell lysis buffer (Cat. No. 11814389001, Roche, Basel, Switzerland) was added to remove the red blood cells. The macrophage (1×10^5^ cells/mL) were then resuspended in RPMI-1640 medium (Cat. No. R8758, Sigma-Aldrich) supplemented with 10% fetal bovine serum and penicillin or streptomycin. The macrophages were plated on the culture dish with a round coverslip for 2 days. After replating the macrophages, samples were used for immunohistochemistry and western blot analyses. For western blot lysate, no specific markers/techniques were used to confirm the purity of macrophages in the sample.

#### Immunohistochemistry

Immunohistochemical staining was performed on the macrophages, which were perfused with 4% paraformaldehyde in PBS (pH 7.2). After fixation, 0.05% NaN_3_ in 0.1 M phosphate buffer was added to the cells. Before immunostaining, slides were incubated in Blocking One Histo (Nacalai, Kyoto, Japan) for blocking the nonspecific binding of antibodies. Subsequently, the cells were incubated with a primary antibody at 4°C overnight. They were then incubated with secondary antibody at room temperature for 2 h in dilution buffer with pH of 7.2 containing 0.01 M phosphate buffer, 0.5 M NaCl, 3% bovine serum albumin, 5% normal goat serum, 0.3% Triton-x100, and 0.05% NaN_3_. The following antibodies were used: (Nedeva, 2021) primary antibodies: anti-nicotinic acetylcholine receptor α7 (rabbit polyclonal, 0.8 mg/mL, Cat. No. ANC-007, 1:100, Alomone Labs, Jerusalem, Israel) and anti-F4/80 (mouse monoclonal, 1.0 mg/mL, Cat. No. NB600-404SS, 1:100, Novus Biologicals, CO, United States) and (Angus and van der Poll, 2013) secondary antibodies: Alexa Fluor 488 highly cross-absorbed goat anti-mouse (Cat. No. A11029, 1:100, Invitrogen, MD, USA) or Alexa Fluor 555 highly cross-absorbed goat anti-rabbit (Cat. No. A21428, 1:100, Invitrogen). After immunostaining, cells were incubated with an autofluorescence quenching kit (Cat. No. SP-8500, Vector laboratories Burlingame CA USA) to suppress autofluorescence. Immunoreactivity was recorded using a Keyence microscope (BZ-X710, Keyence Japan) in optical slice mode; this method is comparable to taking images on a confocal laser microscope. In the immunofluorescence experiment, we observed the field of view using a x10 objective lens. We set regions of interest (ROIs) from 10 cells (usually at least 10 cells were found) within the field of view and quantified how much they changed compared to the CON values. The values of each cell were measured using software and averaged. These procedures were performed for each group of mice, and statistical analysis was conducted. Fluorescence intensity and threshold were determined by Image-J software. Fluorescence intensity and threshold were measured with image-j software. The threshold value was the level at which cell fluorescence could be detected under CON conditions, and the fluorescence intensity in the other groups was compared to the threshold value.

#### Western blot

Western blotting was performed on macrophages and hippocampal tissues. For macrophages, cells (1× 10^5^ cells/mL) from Control, LPS, and LPS + VNS were collected by respective cell scrapers and centrifuged at 400 × g. Regarding hippocampal tissues, the tissues from the mice groups (Bregma from −1.00 mm to −4.00 mm AP) were homogenized with disposable SP and power masher II (Nippi, Inc., Tokyo, Japan).

Pellets from macrophages or hippocampal tissues were lysed in radioimmunoprecipitation assay buffer (Cat. No. 08714–04, Nacalai Tesque, Kyoto, Japan) with 0.1% sodium lauryl sulfate (SDS). The cell lysates were diluted in SDS-containing Laemmli sample buffer (final; 100 mM Tris–HCl, pH 6.8, 4% SDS, 2% 2-mercaptoethanol, 20% glycerol, and 0.01% bromophenol blue) to obtain the final samples (protein concentration, 0.75 mg/mL). After boiling, samples (30 μg) were loaded on 12% SDS-containing polyacrylamide gels (6.25% stacking gel) and separated at 20 mA for 80 min at room temperature using a running buffer (100 mM Tris, 100 mM glycine, and 0.1% SDS). Proteins in the gels were transferred to a polyvinylidene difluoride membrane for 90 min at 115 mA at room temperature. The membranes were blocked in 5% skim milk in 0.05% Tween 20/Tris-buffered saline (TBST) and incubated with antibody at 4°C overnight. The antibodies used were as follows: anti-nAChRα7 (rabbit polyclonal, 0.8 mg/mL, Cat. No. ANC-007, 1:500, Alomone Labs, Jerusalem, Israel), anti-c-fos (rabbit polyclonal, Cat. No. SAB5700610, 1:500, Sigma-Aldrich), anti-ACAD9 (rabbit polyclonal, 0.4 mg/mL, Cat. No. 15770-1-AP, 1:500, Proteintech Group, Inc., IL, United States), anti-TF (mouse monoclonal, 1.0 mg/mL, Cat. No. MAA524Mu21, 1:500, Cloud-Clone Corp., Wuhan, PRC), and anti-actin (mouse monoclonal, Cat. No. A5441, 1:50000, Sigma-Aldrich) antibodies. The membranes were washed three times in TBST and incubated for 1 h with a horseradish peroxidase-conjugated secondary antibody (dilution 1:2,000). Immunoreactive proteins were visualized using the chemiluminescence reagent Luminata (Millipore). Chemiluminescent signals were obtained using ImageQuant LAS-4000 (GE Healthcare).

#### Survival rates

Living or dead mice of the CON (*n* = 6), LPS (*n* = 17), LPS + VNS3h (*n* = 21), LPS + VNS18h (*n* = 12), and LPS + VNS after vagotomy (*n* = 6) groups were recorded in a database for 200 h and analyzed using GraphPad Prism software. Significance was determined using the Kaplan–Meier method.

#### Vagotomy

Vagotomy was conducted in accordance with the procedure described in a previous study (Qian et al., 1999). The right VN in deeply anesthetized mice was surgically isolated and cut. Three days after the operation and during recovery of the operated mice, LPS administration and VNS were used.

#### Nanotag

Body temperature and locomotor activity were measured using Nanotag (Kisse Comtec, Nagano, Japan). Nanotag was embedded s.c. in mice and maintained for 3 days until recovery from surgical operations; following this, the recording was started. The recording parameters were defined as follows: sampling rate of 1 recording/4 min and recorded temperature range of 25°C–37°C.

After recording the baseline parameters for 5 days, LPS was injected intraperitoneally, followed by VNS. After the mice recovered and were examined for 10 days, the Nanotag was collected from mice, and data were transferred to a computed for the analysis.

#### Elisa

IL-1β immunopositive levels in the blood plasma were tested among the mice groups, including the CON, LPS, LPS + VNS3h, and LPS + VNS18h groups, using an ELISA kit (R&D systems, Minneapolis, MN). The experimental procedures were conducted according to the manufacturer’s protocol. Briefly, this experiment was conducted in the following steps: First, blood samples were collected from the mice under deep anesthesia from their left inferior vena cava; Second, in the presence of heparin (1,000 units/mL, Mochida Pharm., Tokyo, Japan), blood suspensions were centrifuged at 400 × g and 4°C for 30 min; Third, the supernatant was applied to a 96-well plate precoated with monoclonal IL-1β antibody; and finally, the measurements of absorbance using 450-nm wavelength light were used to determine the concentration of IL-1β.

#### Electrophysiology: LFPs

Recording electrodes were achieved by soldering tungsten electrodes for the cathode, anode, and reference using a Samtec connector (Unique Medical Co., Osaka, Japan). Under deep anesthesia, the cathode and anode were carefully injected into the hippocampus (bregma −2.00 mm AP), and the reference electrode was placed in the cerebellum. The reference electrode was tightly attached and fixed on the scalp using Exafine (vinyl polysiloxane impression material, GC corp., Tokyo, Japan). LFP recording was performed 2 days after the fixation of the electrodes. Bandpass filters were set as follows: gain, 0.2 mV/V; low-cut filter, 0.5 Hz; and high-cut filter, 120 Hz in a portable electric amplifier (EBA-100, Unique Medical, Co., Osaka, Japan).

#### Proteomics

Mouse hippocampus (weight, 25 mg) was isolated from the CON, LPS, LPS + VNS3h, and LPS + VNS18h groups. First, the protein groups were purified and subjected to liquid chromatography with tandem mass spectrometry (LC–MS/MS) (Supplemental Figure S2). Subsequently, 4,438 protein fragments that were identified using LC–MS/MS were normalized by the GADPH expression levels (Supplemental Figure S2B). Statistical differences among mice were analyzed. The procedures were conducted using the following steps:

(1) Sample Lysis. The tissues were lysed in SDT buffer [4% (w/v) SDS, 100 mM Tris–HCl, 0.1 M DTT, pH = 7.6, concentration sample-to-buffer ratio = 1:10]. Following this, the tissue was homogenized (disposable homogenizer SP and Power Masher II, Nippi, Inc.) and heated at 95°C for 3–5 min. After centrifugation at 16,000 × g for 5 min, the supernatants were used to measure the protein concentration using the BCA assay kit (Pierce Biotechnology, MA, United States).
(2) Sample Processing. Samples for proteomes were prepared using a filter-aided sample preparation method described in a previous study (Wiśniewski et al., 2009). Briefly, the extracted brain homogenates (100 μg) and bovine serum albumin (0.6 μg) were diluted in UA buffer (8 M urea in 100 mM Tris–HCl, pH = 8.5) and placed in a 30 K filter. The following steps were repeated twice: centrifugation at 14,000 × g and addition of flow-through solution to 50 mM iodoacetate in UA buffer. Thereafter, incubation was performed in a dark room at room temperature for 20 min and centrifuged at 14,000 × g. The flow-through solution was added to 50 mM ammonium bicarbonate (AmBic) and centrifuged at 14000 × g (repeat this step twice). The flow-through solution was added to 50 mM AmBiC and 200 ng/μL trypsin/Lys-c mix in 50 mM acetic acid for enzyme digestion. After the incubation of the filter unit at 37°C overnight, 50 mM AmBiC was added to the filter unit and centrifuged at 14,000 × g. Thereafter, 0.5 M NaCl was added and centrifuged at 14000 × g. The samples were acidified with trifluoroacetic acid, and the protein concentration was determined at Abs 280 nm by NanoDrop. Subsequently, the samples were desalted by stage tip C18 (GL science) and redissolved in 2% acetonitrile and 0.1% trifluoroacetic acid; 200 ng of samples were used for LC–MS/MS.
(3) High-performance Liquid Chromatography. Samples (1 μL) in 2% acetonitrile and 0.1% trifluoroacetic acid were injected into EASY-nLC1000 systems (Thermo Fisher Scientific). The columns used were NANO-HPLC capillary column C18 of 0.075 × 150 mm (Nikkyo Technos). The temperature was set at 45°*C. mobile* phases were set at 0.1% formic acid and acetonitrile/0.1% formic acid. The gradient was set at 0/5–100/30–120/65 (min/%B) and 120 min for the total time. The flow rate was set at 300 nL/min. The trap columns used were Acclaim PepMap 100 precolumns of 0.1 × 20 mm (Thermo Fisher Scientific).
(4) Mass Spectroscopy. The samples were applied to a Q Exactive Mass Spectrometer (Thermo Fisher Scientific). The set parameters were defined as follows: method, Top 10 method; spray voltage, 2,300 V; capillary temperature, 275°C; mass range, 350–1800 m/z; and normalized collision energy, 28%.
(5) Database Search. Data were analyzed using Proteome Discoverer (ver. 2.2) using the Mascot search engine (Ver. 2.6.0). Conditions for the analyses were set as follows: database, SwissPlot 2018_8; taxonomy, *Mus musculus* (house mouse); enzyme, trypsin; precursor mass tol., 6 ppm; fragment mass tol., 20 mmu; dynamic modification, oxidation (M); and static modification, carbamidomethyl (C).

**Figure 1:**
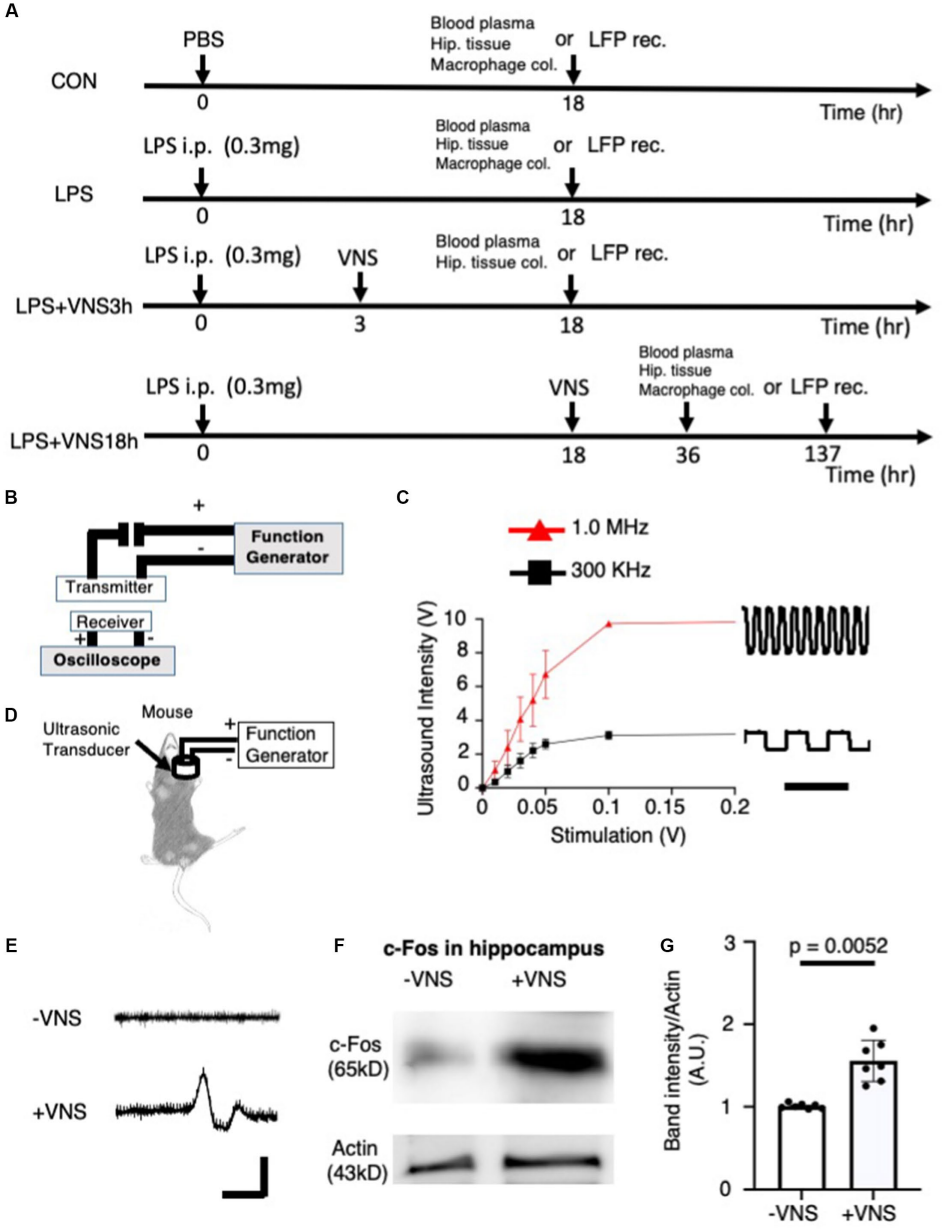
Electrical excitability of cervical vagal nerve (VN) and hippocampal neurons by ultrasound stimulation. **(A)** Experimental procedure of ultrasound vagal nerve stimulation (VNS). **(B)** The detector of ultrasound for detecting and analyzing ultrasonic wave. **(C)** Input–output relationship of ultrasound devices. Bar: 5 μs. **(D)** Stimulation of vagus nerve by ultrasound in mice. **(E)** Recording of action potential in vagal nerve. No gel was existed between the detector and the electrode. Bar: 2 ms, 10 μV. **(F)** Increased c-fos (a marker for the excitability of neurons) in the hippocampus after ultrasound VNS. Tissues were collected at 2 h after VNS. Immunoreactive band with anti-actin antibody for control. **(G)** Statistical analysis of c-fos expression per CON in the hippocampus before and after VNS for 14 mice (-VNS: 7 mice and + VNS: 7 mice). Significance (*p* < 0.05) was determined between the no stimulation of vagal nerve (−VNS) and stimulation of vagal nerve (+VNS) groups using the Student’s t-test.

#### Statistics

Results were expressed as mean ± standard error of the mean. For proteins, normality was tested based on the expression levels of Actin. Two sets of data were analyzed using the Student’s *t*-test. Multiple comparisons were conducted using one-way analysis of variance, followed by Tukey’s post-test. A value of p of <0.05 was considered significant. For proteomics, statistics were performed by the following steps: (1) the detected protein abundances were normalized by GADPH abundance (Supplemental Figure S1), (2) the proteins that satisfy the following conditions were listed (abundance counts are greater than or equal to “3” in every sample and abundance (grouped) CV(%) were less than or equal to “20” in every sample group and abundance ratio variabilities (%) were less than or equal to “30” in every ratio.), 403 candidate proteins were obtained, and (3) The proteins that satisfy the following conditions were listed (value of *p* <0.05 and log2 (LPS/ CON ratio) > 0.2). 12 candidate proteins were obtained. Data were analyzed using GraphPad Prism 9.0 software (San Diego, CA, United States).

## Results

### Noninvasive ultrasound VNS

In this study, the SE mouse model was used and ultrasound was applied to the mice according to the line of experiments (Table 1) and experimental timeline shown in Figure 1A. Before the mouse experiments, the ultrasound was characterized with respect to the detector. When the ultrasonic transducer was connected to a function generator that can change the frequency of the ultrasonic wave (Figure 1B), the ultrasound intensity revealed a linear input–output relationship for voltage-dependent ultrasound intensity and 1 MHz was found to transmit more intensity than 300 kHz (Figure 1C).

**Table 1:** Summary of Methodology.

Figure number	Methodology	Number of mice
Figure 1	Proteomic experiment of hippocampal tissue *ex vivo*	8
Figure 2	Experiment1: Characterization of ultrasound and VNS Experiment2: VNS for mice and its effect on neuron *in vivo*	17
Figure 3	Peritoneal macrophage *ex vivo*	21
Figure 4	Plasma interleukin-1 beta *ex vivo*	20
Figure 5	Survival rate *in vivo*	62
Figure 6	Hypothermia and locomotor activity *in vivo*	12
Figure 7	Recording of hippocampal brain activity *in vivo*	9
Figure 8	Proteomic and immunoblot analyses of hippocampal tissue *ex vivo*	20
Supplemental Figure S1	Preparation of proteomic experiment *ex vivo*	8
Supplemental Figure S2	Electrocardiogram recording *in vivo*	8
Supplemental Figure S3	Temperature *in vivo*	9

**Figure 2:**
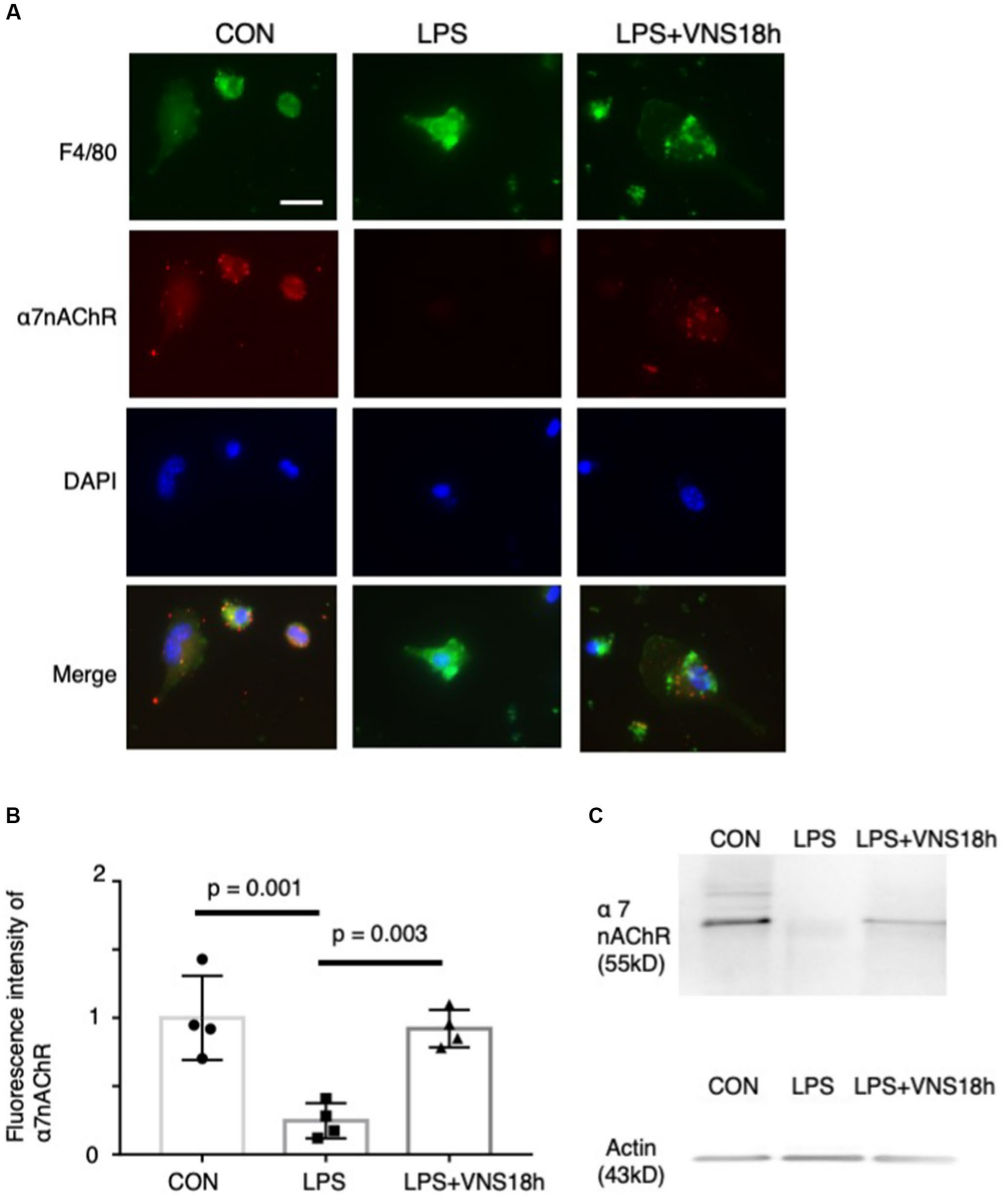
Dynamics of **α**7 nicotinic acetylcholine receptor (α7nAChR) expression in peritoneal macrophages by lipopolysaccharide (LPS) and ultrasound stimulation of VN. Reduced α7nAChR immunoreactivity after LPS administration and recovery of the signals after additional VNS are presented. **(A)** Immunoreactivity of F4/80 (1st row) and α7nAChR (2nd row) antibodies. Macrophages were isolated from mice as follows: CON mice, 18 h after i.p. injection of phosphate-buffered saline (PBS); LPS mice, 18 h after i.p. injection of 0.3 mg LPS; and LPS + VNS18h, 18 h after i.p. injection of 0.3 mg LPS and VNS administration. **(B)** Statistical analysis of α7nAChR immunoreactivity in macrophages. Each dot: average value from each mouse (*n* = 4 mice for each group; *p* < 0.05: significant). **(C)** Western blot analysis of macrophages from mice groups using anti-α7nAChR (*n* = 3 mice for each group). Immunoreactive band with anti-actin antibody for control.

Ultrasound stimulation in the brain evokes neuronal activity (Tufail et al., 2011). The ultrasound transducer, which was connected to the function generator, was placed on the neck muscle (Figure 1D). Next, we tested whether ultrasound VNS at 1 MHz evokes action potentials (APs), we recorded the electrical excitability of neurons from VN (Figure 1E). The recording electrode was injected to the VN fiber and APs were recorded (Figure 1E). Without VNS, no APs occurred (Figure 1E upper panel), but when VNS was added, action potentials were observed (Figure 1E lower panel), suggesting that VNS activates electrical activity in the vagal nerve.

Subsequently, we examined whether VNS effectively modulated hippocampal neuronal activity. The hippocampal tissues were isolated, and the c-fos (i.e., a marker for neuronal depolarization (VanElzakker et al., 2008) immunoreactivity) was compared between the −VNS and + VNS groups. Notably, the expression level of c-fos was higher in the +VNS group than in the −VNS group (Figures 1F,G). These findings suggest that VNS stimulates hippocampal neurons and evokes action potential.

### Recovery of α7nAChR expression in macrophages by VNS

To test whether the noninvasive stimulation of the bilateral cervix of mice changes the immune level, we isolated peritoneal macrophages from mice after lipopolysaccharide (LPS) administration and conducted VNS 18 h later; subsequently, we immunostained them with an anti-α7nAChR antibody (Figure 2). F4/80 (i.e., a marker for macrophages)-positive cells exhibited α7nAChR immunoreactivity (Figure 2A), suggesting that α7nAChR was expressed in peritoneal macrophages. Subsequently, the effects of LPS administration and VNS were examined. α7nAChR immunoreactivity was significantly reduced in macrophages after LPS administration (*p =* 0.001, CON vis-à-vis LPS, *n* = 4 mice, Figures 2B,C). However, VNS recovered the α7nAChR immunoreactivity to the CON level (*p* = 0.003, LPS vis-à-vis LPS + VNS18h (i.e., VNS at 18 h after administration), *p* = 0.857, CON vis-à-vis LPS + VNS18h, *n* = 4 mice, Figures 2B,C). These findings suggest that LPS administration reduces α7nAChR function and VNS recovers it.

**Figure 3:**
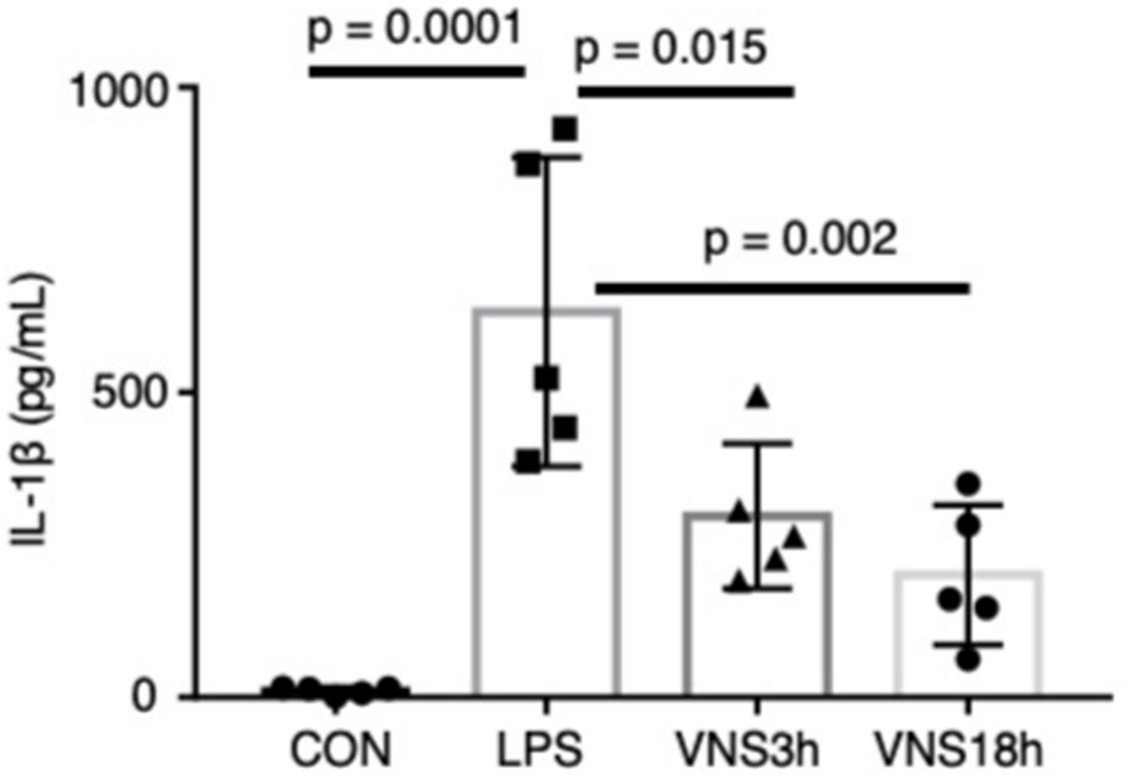
Dynamics of plasma interleukin-1β after the administration of LPS and VNS in mice. IL-1βvalue from each mouse group. *n* = 5 mouse in each group. Statistical significance was determined by one-way ANOVA followed by Tukey’s post-test. *p* < 0.05: statistical significance.

### Recovery of the plasma interleukin (IL)-1β level after VNS

To test whether VNS alleviates SE, the plasma level of IL-1β was measured using enzyme-linked immunoassay (ELISA) (Figure 3). Similar to the previous findings, LPS administration significantly elevated the plasma IL-1β level (Figure 3; CON, 10.77 ± 2.83 pg./mL; LPS, 631.9 ± 113.1 pg./mL, *p =* 0.0001, *n* = 5 mice). Both LPS + VNS3h (i.e., VNS at 3 h after LPS administration) and LPS + VNS18h produced IL-1β against the LPS group (Figure 3; LPS + VNS3h, 297.4 ± 53.1 pg./mL, *p =* 0.014, *n* = 5 mice; LPS + VNS18h, 200.9 ± 51.1 pg./mL, *p =* 0.018, *n* = 5 mice). These findings suggest that VNS significantly reduced cytokine levels after SE induction.

**Figure 4:**
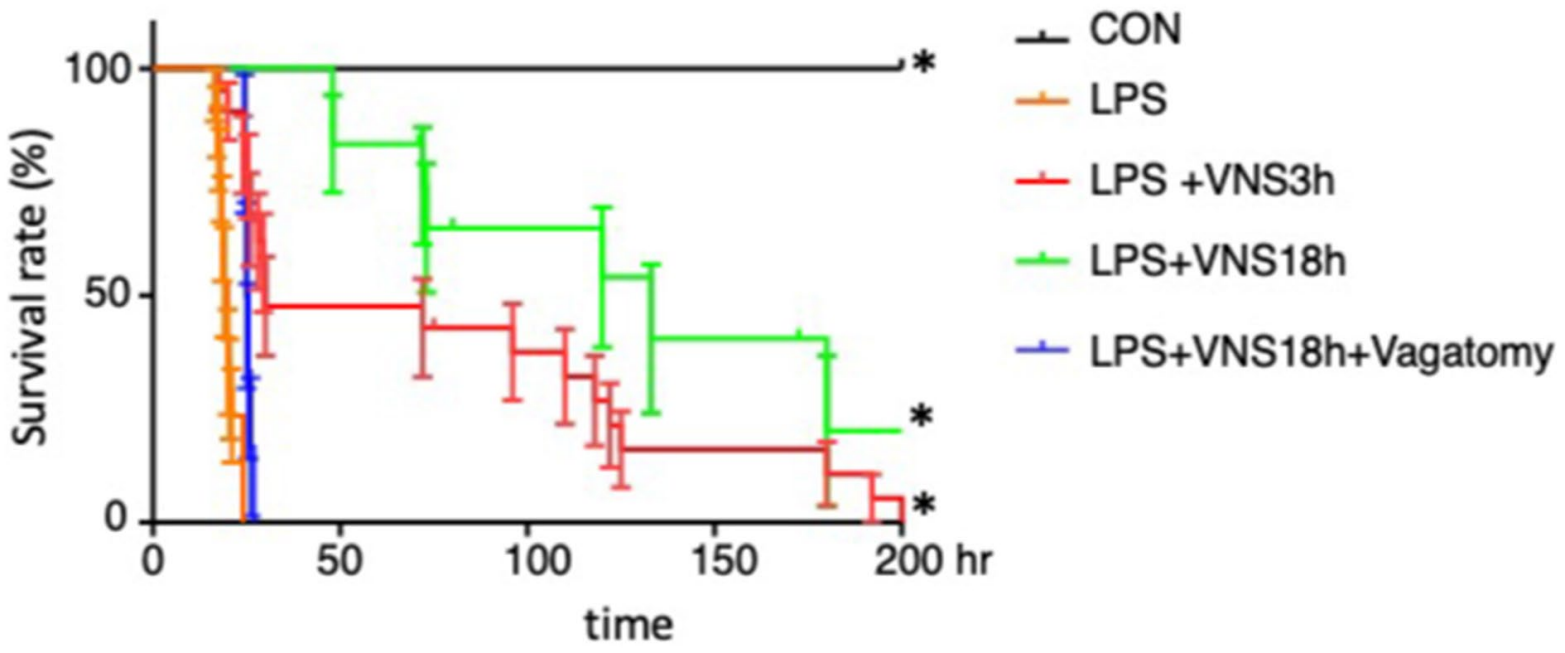
Lethal survival rate after LPS and its recovery in association with VNS. The survival rates were analyzed by Kaplan-Meyer method and compared using a log-rank test. *p* < 0.05 was determined as statistical significance. The number of mice used for statistical processing is as follows: *n* = 6 (CON), 17 (LPS), 21(LPS + VNS3h), 12 (LPS + VNS18h), 6 (LPS + VNS18h + Vagotomy).

### Improvement in survival rates by VNS

Moreover, we examined whether VNS potentially improves the survival rates within 200 h in SE. The survival rates of the CON, LPS, LPS + VNS3h, LPS + VNS18h, and LPS + VNS18h + vagotomy groups are shown in Figure 4. Compared with LPS, LPS + VNS3h and LPS + VNS18h improved the survival rates. However, LPS + VNS18h + vagotomomy (i.e., LPS and VNS18h treatment after unilateral vagotomy) nullified the improvement in the survival rates. These findings suggest that VNS is able to improve survival rate after SE.

**Figure 5:**
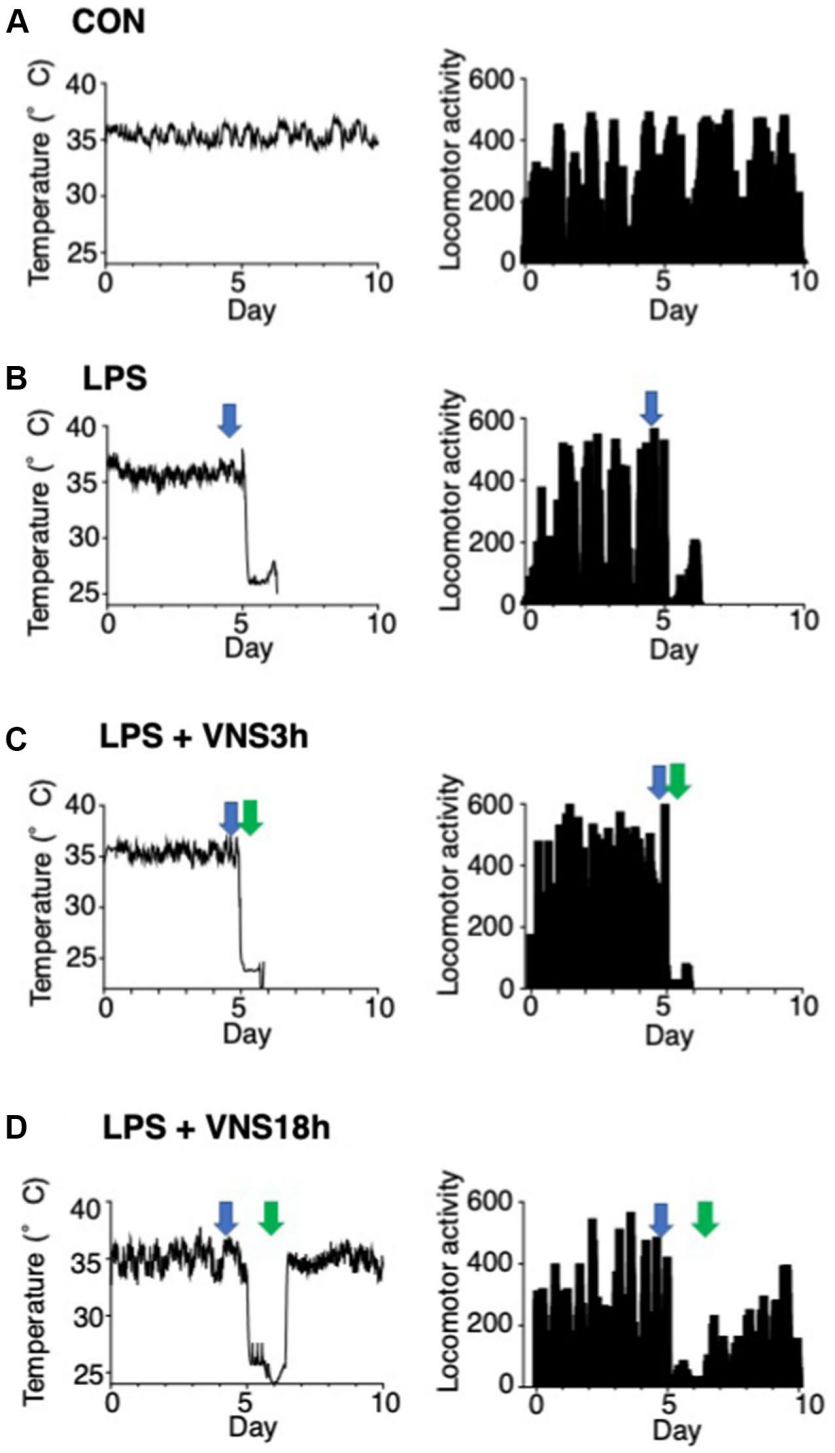
Hypothermia and ablation of locomotor activity after LPS and their recovery after VNS18h. **(A–D)** Time course changes in body temperature and locomotor activity. Left panels: subcutaneous abdominal body temperature. Right panels: spontaneous locomotor activity. Arrows indicate the timing of injection or VNS: open arrows, PBS injection; blue arrows, LPS injection; green arrows: VNS. Recordings were performed for 10 days at three time points: (Nedeva, 2021) at baseline for 5 days, (Angus and van der Poll, 2013) at LPS administration after 0.5 h of baseline recording, (Gofton and Young, 2012) at VNS after 3 h of LPS administration (i.e., VNS3h), (Imamura et al., 2011) at VNS after 18 h of LPS administration (i.e., VNS18h). **(A)** CON mice, **(B)** LPS-treated mice, **(C)** LPS + VNS3h-treated mice, **(D)** LPS + VNS18h-treated mice (*n* = 3 mice in each group).

### Alleviation of body temperature and locomotor activity by VNS after SE induction

Body temperature and locomotor activity were recorded using Nanotag, and the results are shown in Figure 5. The body temperature of mice was stable in the CON group (Figure 5A). However, the temperature of the other groups decreased, and locomotor activities were drastically reduced after LPS administration (Figures 5B–D). In contrast, VNS3h, i.e., VNS at 3 h after LPS administration, failed to provide temperature and locomotor activity recovery (Figure 5C), whereas VNS18h rapidly induced recovery in temperature and locomotor activity (Figure 5D). These findings suggest that VNS18h improved the condition of the SE mice.

**Figure 6:**
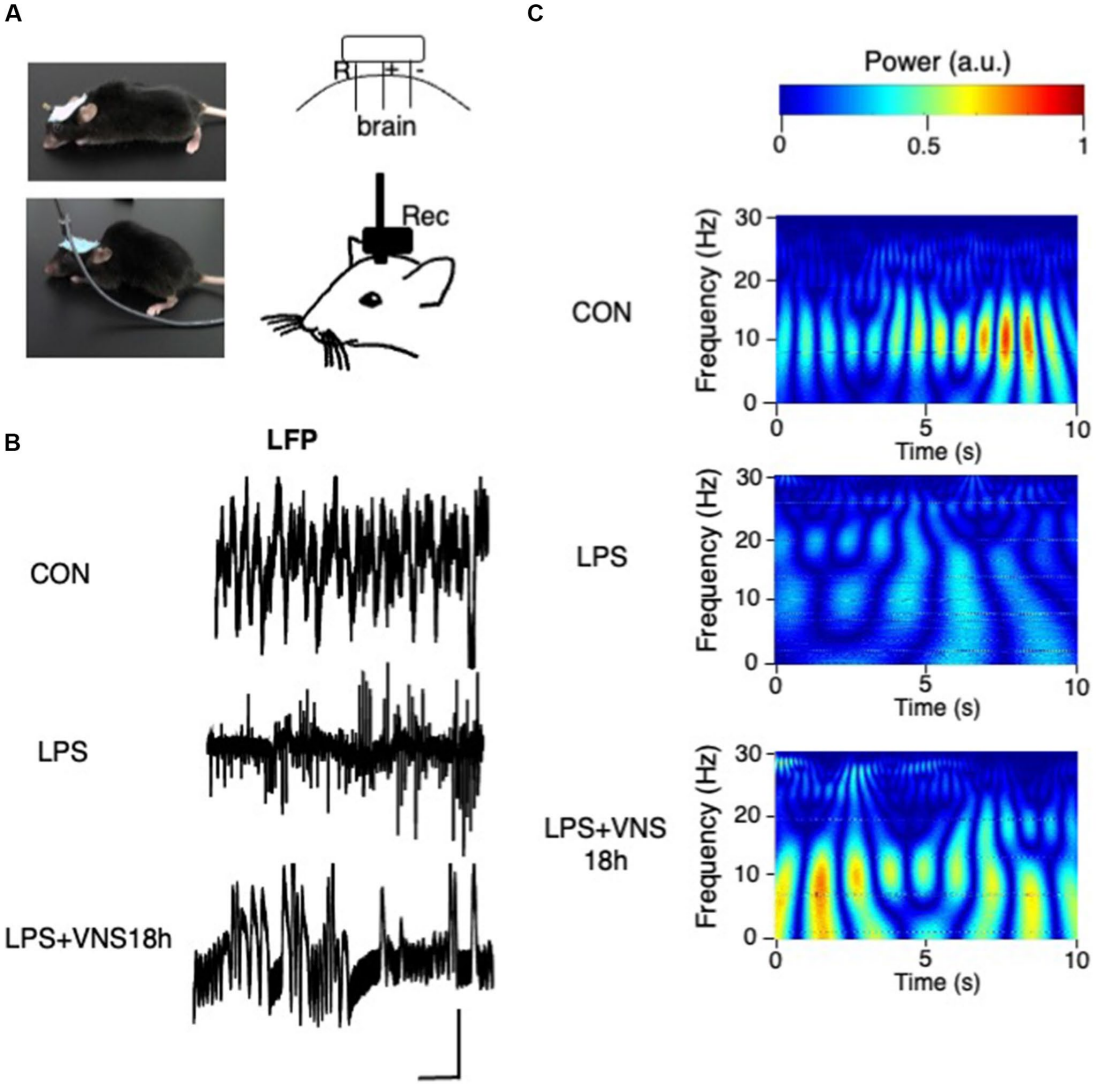
Aberrant hippocampal brain activity after LPS and their recovery after VNS18h. **(A)** Experimental setting for LFP recording. **(B)** Traces of LFP recordings. Bar: 1 mV, 1 s. **(C)** Wavelet power spectrum. *n* = 3 mice in each group.

### Recovery of hippocampal brain activity pattern by VNS after SE induction

To examine whether VNS improved the hippocampal brain activity in SE, the electrode for recording local field potentials (LFPs) for hippocampal neuronal activities was attached in the mouse brain (Figure 6A). Although no difference was found in the amplitude of LFP (Figure 6B), the results of wavelet analyses showed that the activity patterns around 10 Hz were clearly visible in CON (Figure 6C, top panel), whereas they disappeared in LPS (Figure 6C, middle panel) and recovered again in LPS + VNS18h (Figure 6C, bottom panel). These findings suggest that VNS recovers the altered hippocampal brain activity patterns around 10 Hz after SE.

**Figure 7:**
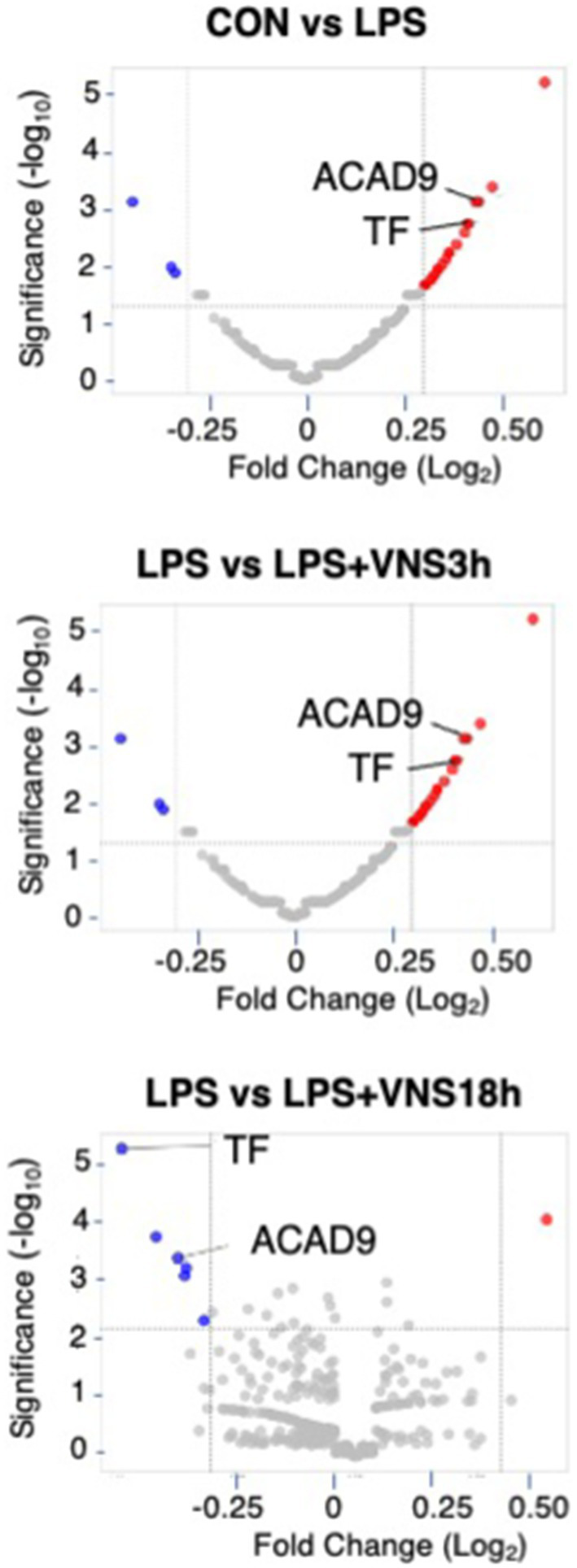
Proteomic analysis after LPS and VNS. **(A)** Experimental procedure for the preparation of samples. **(B)** Alteration of expression patterns of proteins after VNS against LPS treated groups. TF and ACAD9 were shown as statistical differences. Statistical significance was determined as *p* < 0.05.

### Novel marker proteomic analyses for VNS-induced functional recovery in SE

We hypothesized that candidate protein biomarkers critical for SE induction and functional recovery might increase or decrease after LPS administration and should recover to baseline levels by VNS.

**Figure 8:**
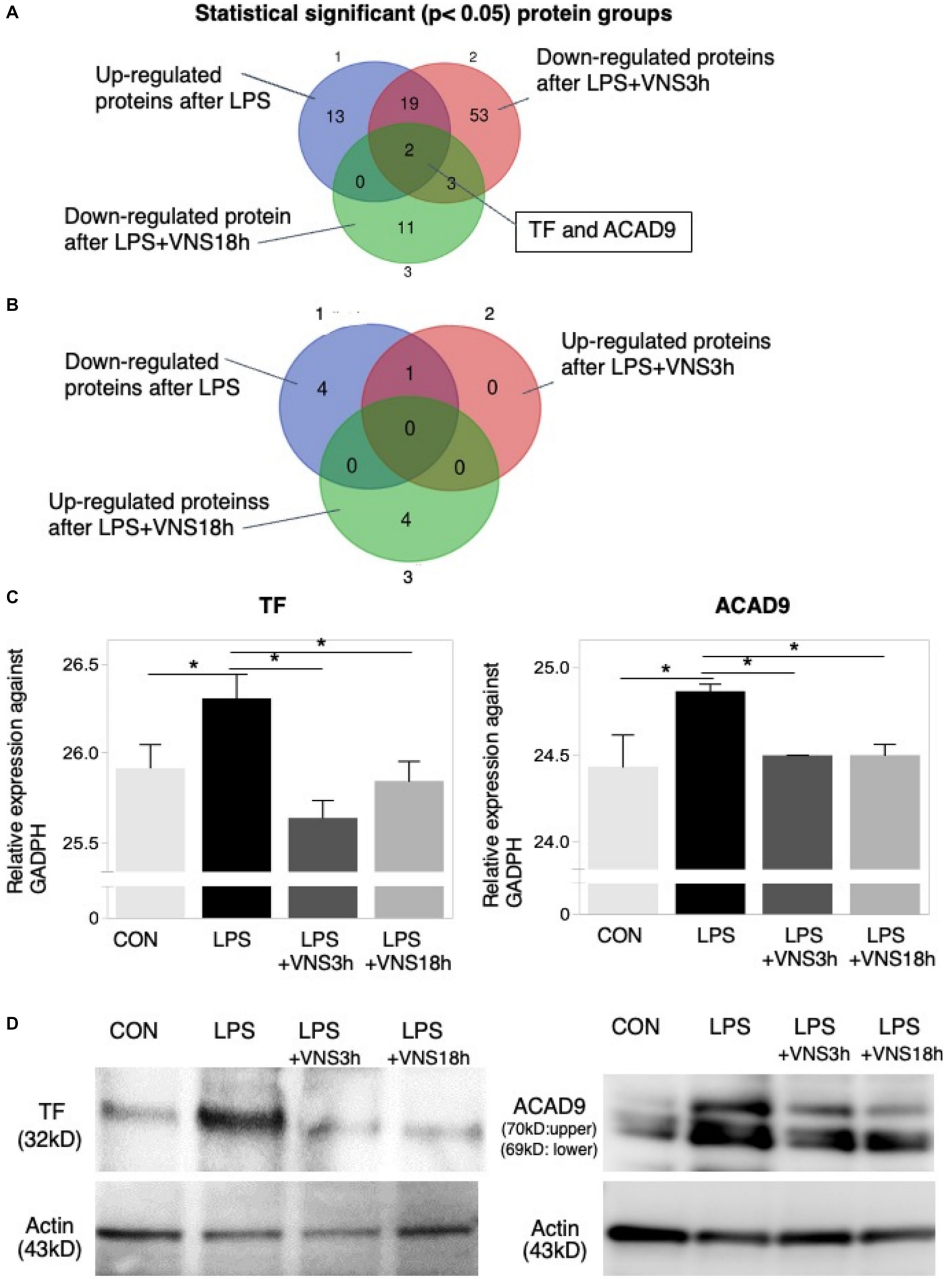
Dynamics of TF and ACAD9 expression after LPS and VNS. **(A,B)** Statistical significant protein groups after LPS and VNS from proteomic analysis of hippocampal tissues. Groups A and B have detected opposite sets of proteins. **(C)** Expression level of TF (left panel) and ACAD9 (right panel) against GADPH. **(D)** Western blot analysis (*n* = 3 mice in each group) using anti-TF (left panels) and anti-ACAD9 (right panels). The bottom panels indicate the western blotting using anti-actin.

To do this, we performed HPLC and LC–MS analysis with hippocampal tissue (Supplemental Figure S1). Based on the dynamics of protein expression levels for the novel biomarker in SE (Figure 7 and Supplemental data 1), we analyzed the three groups for comparison: (1) CON vs. LPS for proteins related to SE pathogenesis (Figure 7, top panel), (2) LPS vs. LPS + VNS3h for proteins related to recovery from acute systemic inflammation (Figure 7, middle panel), and (3) LPS vs. LPS + VNS18h for proteins related to recovery from SE (Figure 7, bottom panel). Out of the total proteins, 13 were upregulated after LPS, 53 were downregulated after LPS + VNS3h, and 11 were downregulated after LPS + VNS18h. However, the expression level of acyl-CoA dehydrogenase family member 9 (ACAD9) and tissue factor (TF) (CD142) exclusively increased after LPS administration and recovered to the baseline level after VNS (Figures 8A–C).

To evaluate reproductivity, western blotting using anti-ACAD9 and anti-TF was performed on the mice groups. Both expression levels were similar to the proteomic results (Figure 8D). These findings suggest that ACAD9 and TF are useful biomarkers for SE.

### Evaluation of safety for VNS

Finally, we examined whether VNS aggravates the body condition. Electrocardiogram was used to evaluate the mice groups before and after VNS. No difference in the electrocardiogram was found between the two groups (Supplemental Figure S2A). In contrast, heart rates after VNS were slower than those before VNS (Supplemental Figure S2B). We also compared the skin/body temperatures before VNS, after VNS with 50% duty, and after VNS with 50% duty. No significant difference was found between these mice groups (Supplemental Figure S3). The previous findings suggest that VNS is a noninvasive therapy that leads to no harmful effects on SE mice.

## Discussion

### Noninvasive ultrasound VNS in an SE mouse model

This study aimed to develop a noninvasive method for the improvement of survival rate and recovery of brain dysfunction in SE. Ultrasound examination at 1 MHz successfully and noninvasively activated the VN electrical excitability. Previous studies have suggested that ultrasound induces the electrical excitability of neurons *via* the activation of mechanosensitive ion channels (Ye et al., 2018; Qiu et al., 2020). We found that VNS by ultrasound induced the electrical excitability of VN fibers and significantly activated c-fos expression in the hippocampus; notably, c-fos is a marker for the electrical excitability of neurons after nerve fiber stimulation (Joo et al., 2016).

Several studies have reported that VN activation modulates hippocampal function (Theodore and Fisher, 2004; Suarez et al., 2018). Consistent with the findings of the previous reports, our findings reported upregulated c-fos expression levels in the hippocampus after VNS (Shin et al., 2019). To the best of our knowledge, this is the first study to demonstrate a novel therapeutic potential of VNS for SE. Previous reports have indicated that the electrical excitation of VN improves the recognition memory (Clark et al., 1999) as well as alleviates epilepsy (FineSmith et al., 1999), cluster headache (Wei and Goadsby, 2021), and mental illness (Carpenter et al., 2004). Therefore, VN activation exhibits therapeutic effects on brain hypofunction. Clinical significance of VN is reported as brain functional recovery in patients for epilepsy, depression, headache, ischemic stroke (Goggins et al., 2022).

### Improvement of survival rates and recovery of α7nAChR in macrophages after VNS in an SE mouse model

We reported that VNS critically improved the survival rates in an SE mouse model. This finding may be related to the clinical data for improvement of survival rate using VNS (Teton et al., 2019). Some studies have reported that the cholinergic anti-inflammatory pathway controlled the innate immune response and that VN is critically involved in this mechanism (Borovikova et al., 2000; Wang et al., 2003).

Regarding the innate immune response, we determined whether peritoneal macrophages were activated by VNS in an SE mouse model. We found that macrophages from the abdominal cavity reduced the immunoreactivities of α7nAChR after LPS and induced recovery of its expression after VNS. The α7nAChR expressed in the macrophages is critical for controlling the immune response (Rosas-Ballina et al., 2011). Our findings report an effective control of macrophages by ultrasound VNS in SE. For α7nAChR, the dominant negative regulator—dupa 7 isoform—regulates the surface expression of α7nAChR, and this may be involved in reduced α7nAChRs in LPS-treated macrophages (Maldifassi et al., 2018).

### Recovery of body temperature, locomotor activity, and hippocampal activity pattern in an SE mouse model using ultrasonic VNS

Body temperature is aberrant in patients with sepsis. Hypothermia also occurs in patients with sepsis (Hotchkiss et al., 2016) and patients with encephalopathy after systemic inflammatory response syndrome (Magalhães et al., 2015). We found that the body temperature of mice was critically reduced after SE induction and that it gradually recovered after VNS administration in an SE mouse model. However, body temperature recovery was obscure when VNS was administered 1 h after LPS administration. The body temperature was mainly controlled by the neural system, including the hypothalamus (Satinoff, 1978). However, the hypothalamus in circumventricular organs is mainly vulnerable to BBB dysfunction in SE (Sisó et al., 2010). Therefore, the aberrant activity patterns of hippocampal neurons may contribute to alterations in hypothalamic neuronal activities, and this, in turn, results in the dysregulation of body temperature. Moreover, the locomotor activity was diminished in SE, but it was involved in hippocampal activity patterns (Purandare et al., 2022). Our findings indicate that the hippocampal activity patterns were aberrant in an SE mouse model and are similar to those of previous reports, and the recovery of locomotor activity after ultrasound stimulation may be involved in the recovery of hippocampal activity patterns.

### Novel biomarkers in an SE mouse model

Proteomics analysis is a powerful tool for identifying novel biomarkers. However, data on proteomics, particularly those relevant to the pathogenesis and recovery phases, targeting SE are still lacking. First, we demonstrated two functional proteins, namely TF and ACAD9, whose expressions were increased upon LPS administration and recovered to normal levels after VNS. TF is a protein encoded by the F3 gene that is present in leukocytes and plays a role in the initiation of thrombin formation. Because thrombin formation initiates disseminated intravascular coagulation (Niessen et al., 2008), which is often observed in SE patients (Blauhut et al., 1985; Pérez-Fraile and González-Elipe, 1986), the TF is a candidate marker for SE. Moreover, ACAD9 was found to be another biomarker for SE. ACAD9 is an assembly factor for the mitochondrial respiratory chain complex 1 (Scheffler, 2010). ACAD9 levels are altered in mitochondria-related diseases, including mitochondrial encephalomyopathy (Garone et al., 2013) and cardiomyopathy (Sinsheimer et al., 2021). ACAD9 proteins are abundantly expressed in the brain (He et al., 2007). Severe symptoms related to ACAD9 include edema, coma, and cognitive dysfunctions (Li et al., 2015). These symptoms are similar to those of SE. Overall, focusing on the expression levels of TF and ACAD9 is potentially useful in the evaluation of SE severity; however, the molecular mechanism requires further evaluation.

### Biosafety and side effects of ultrasound VNS

The imbalance between sympathetic and parasympathetic vagal activities is often lethal (Jouven et al., 2005). According to our findings, stable electrocardiography before and after VNS suggests that there were no adverse effects of ultrasound VNS.

Ultrasound often affects the temperature of the illuminating region. However, the lower duty cycle (<60%) does not affect the temperature (Manuel et al., 2020). We used a 50% duty cycle, and no effect on body temperature was observed in mice. Our results suggest that the therapeutic effect of VNS does not involve thermogenesis.

### Study limitations

This study has some limitations. First, LPS is a molecule that plays a significant role in the pathophysiology of sepsis and, when combined with appropriate study design, can provide valuable insights. We initially attempted to use the CLP model for our experiments. However, in this study, we had to perform surgical procedures that were involved in the measurements in several experiments. Considering the additional surgical intervention and the concern that CLP induction experiments could cause excessive damage to the mice, we chose to conduct our research using the LPS model. However, using a more translational model such as CLP (cecal ligation and puncture), testing effect of VNS warrant further evaluation. Second, some studies suggest that the systemic inflammatory response to sepsis may affect the central nervous system, causing changes in inflammatory cytokines and neurotransmitters (Semmler et al., 2008; Pavlov and Tracey, 2017). On the other hand, the level of peripheral and central nervous system inflammation may not be directly affected (Barichello et al., 2005). Further research is needed to clarify whether the increased inflammation in the periphery may or may not be directly related to central inflammation. Third, this study focused only on the hippocampus, but given that SE may also affect the frontal lobe, it is necessary to consider the frontal lobe in the future. Third, VNS may affect microglia. Appropriate VNS may suppress microglial inflammatory responses and promote neuroprotective and repair processes In addition, VNS also affects neurotransmitter balance. Therefore, proper VNS may regulate neurotransmitter balance and contribute to neuroprotection and repair and is worth considering. Fourth, we performed unilateral vagotomy in this study, and previous experiments involving unilateral vagotomy showed survival for more than 2 days, indicating that unilateral vagotomy in mice does not significantly affect survival rates. However, considering the immune physiology role of the vagal nerve, it is important to consider the potential impact of vagotomy itself on survival rates in the future study. Fifth, no sample calculation was performed and most of the parameters were examined in the laboratory, and it is necessary to determine whether these parameters are sufficient to reflect clinical improvement. Hence, it is important to highlight the remaining gaps, such as ultrasound intensity, brain activity measurements, temperature, and locomotor activity, which should be addressed in further studies to overcome the current limitations.

### Advantages of ultrasound VNS compared to invasive methods

We will discuss the advantages of ultrasound VNS in comparison to invasive VNS methods as well as other non-invasive VNS techniques. The ultrasound VNS offers four distinct advantages over invasive VNS methods: (1) non-invasiveness: Unlike invasive VNS techniques that require surgical implantation of electrodes, ultrasound VNS is non-invasive, eliminating the need for invasive procedures. This non-invasiveness reduces the risk of complications associated with surgical implantation, such as infection, lead migration, and tissue damage (Tufail et al., 2011). (2) absence of surgical implantation: With ultrasound VNS, there is no need for surgical implantation of electrodes, thereby minimizing the associated surgical risks and allowing for a simpler and more convenient procedure. This feature also facilitates repeated stimulation sessions without the need for electrode repositioning or replacement. (3) Reduced risk of infection: Invasive VNS methods carry the inherent risk of infection due to the presence of implanted devices (Ben-Menachem et al., 2015). In contrast, ultrasound VNS eliminates the risk of infection associated with surgical implantation, as it is a non-invasive external stimulation technique. (4) Potential for targeted stimulation: Ultrasound VNS enables precise targeting of specific neural structures by adjusting the focus and intensity of the ultrasound beam (Legon et al., 2018). This targeted stimulation has the potential to improve the specificity and effectiveness of VNS therapy, minimizing off-target effects and optimizing therapeutic outcomes.

### Comparison of ultrasound VNS with other non-invasive VNS techniques

There are various non-invasive VNS techniques. We will compare ultrasound VNS with other non-invasive VNS methods, addressing their specific advantages and highlighting the unique benefits of ultrasound VNS. (1) Transcutaneous VNS: The transcutaneous VNS, utilizing external electrodes placed on the skin, has been explored as a non-invasive alternative (Song et al., 2023). However, ultrasound VNS offers the advantage of deeper tissue penetration (Deffieux et al., 2013), allowing for stimulation of targeted structure that may be difficult to reach with transcutaneous approaches. (2) Magnetic VNS: The magnetic VNS utilizes magnetic fields to induce neural modulation. While it shares the non-invasiveness of ultrasound VNS, ultrasound offers the advantage of higher spatial resolution, enabling more precise and localized stimulation. (3) Trigeminal nerve stimulation: The trigeminal nerve stimulation is another non-invasive VNS technique that targets the trigeminal nerve. The ultrasound VNS, with its potential for targeted stimulation, provides an alternative approach that can directly stimulate specific brain regions with greater precision.

## Conclusions: ultrasound VNS

Ultrasound is a useful and noninvasive method for VNS. As VNS is critical in activating cholinergic anti-inflammatory signaling and ameliorating SE, our results suggest that activating cholinergic anti-inflammatory signaling is a potentially valid target for the novel therapeutic strategies for SE. In addition, TF and ACAD9 proteins are important in SE pathogenesis and recovery. In conclusion, VN activation using ultrasound will be a powerful tool for the active therapeutic intervention of SE.

## Data availability statement

The datasets presented in this study can be found in online repositories. The names of the repository/repositories and accession number(s) can be found in the article/Supplementary material. The dataset used in this study are available at https://doi.org/10.6084/m9.figshare.21721433.v1. These Source data files are provided as Supplementary Data 1.

## Ethics statement

The animal study was reviewed and approved by Committee of Doshisha University (A21011) and Osaka University Medical School (25-094-000).

## Author contributions

YI considered the experimental design and performed all experiments. HM participated in proteomic analyses. JI developed the ultrasound stimulation. KY and NY contributed to the establishment of the experimental system for VNS. YM, SM, JN, and TY performed the statistical analyses of experimental data. YI, NM, HO, JO, and TS discussed the results and supervised this project. All authors contributed to the article and approved the submitted version.

## Funding

This work was supported by grants from Grant-in Aid for Scientific research from the Japan Society for the Promotion of Sciences (24592734, 17H04364 and 20K21587).

## Conflict of interest

JI were employed by Molex corporation.

The authors declare that this study received funding from the Japan Society for the Promotion of Sciences (24592734, 17H04364 and 20K21587). The funder was not involved in the study design, collection, analysis, interpretation of data, the writing of this article or the decision to submit it for publication.

The remaining authors declare that the research was conducted in the absence of any commercial or financial relationships that could be construed as a potential conflict of interest.

## Publisher’s note

All claims expressed in this article are solely those of the authors and do not necessarily represent those of their affiliated organizations, or those of the publisher, the editors and the reviewers. Any product that may be evaluated in this article, or claim that may be made by its manufacturer, is not guaranteed or endorsed by the publisher.

## References

[ref1] Angus D. C. van der Poll T. (2013). Severe Sepsis and septic shock. N. Engl. J. Med. 369, 840–851. doi: 10.1056/NEJMra1208623 23984731

[ref2] Barichello T. Martins M. R. Reinke A. Feier G. Ritter C. Quevedo J.. (2005). Cognitive impairment in sepsis survivors from cecal ligation and perforation. Crit. Care Med. 33:221-3; discussion 62, 1671–1223. doi: 10.1097/01.CCM.0000150741.12906.BD, PMID: 15644673

[ref3] Ben-Menachem E. Revesz D. Simon B. J. Silberstein S. (2015). Surgically implanted and non-invasive vagus nerve stimulation: a review of efficacy, safety and tolerability. Eur. J. Neurol. 22, 1260–1268. doi: 10.1111/ene.12629, PMID: 25614179 PMC5024045

[ref4] Blauhut B. Kramar H. Vinazzer H. Bergmann H. (1985). Substitution of antithrombin III in shock and DIC: a randomized study. Thromb. Res. 39, 81–89. doi: 10.1016/0049-3848(85)90123-9, PMID: 4035650

[ref5] Borovikova L. V. Ivanova S. Zhang M. Yang H. Botchkina G. I. Watkins L. R.. (2000). Vagus nerve stimulation attenuates the systemic inflammatory response to endotoxin. Nature 405, 458–462. doi: 10.1038/35013070, PMID: 10839541

[ref6] Carpenter L. L. Moreno F. A. Kling M. A. Anderson G. M. Regenold W. T. Labiner D. M.. (2004). Effect of vagus nerve stimulation on cerebrospinal fluid monoamine metabolites, norepinephrine, and gamma-aminobutyric acid concentrations in depressed patients. Biol. Psychiatry 56, 418–426. doi: 10.1016/j.biopsych.2004.06.025 15364040

[ref7] Chung H.-Y. Wickel J. Brunkhorst F. M. Geis C. (2020). Sepsis-associated encephalopathy: from delirium to dementia? J. Clin. Med. 9:703. doi: 10.3390/jcm9030703, PMID: 32150970 PMC7141293

[ref8] Clark K. B. Naritoku D. K. Smith D. C. Browning R. A. Jensen R. A. (1999). Enhanced recognition memory following vagus nerve stimulation in human subjects. Nat. Neurosci. 2, 94–98. doi: 10.1038/4600 10195186

[ref9] Deffieux T. Younan Y. Wattiez N. Tanter M. Pouget P. Aubry J. F. (2013). Low-intensity focused ultrasound modulates monkey visuomotor behavior. Curr. Biol. 23, 2430–2433. doi: 10.1016/j.cub.2013.10.029, PMID: 24239121

[ref10] FineSmith R. B. Zampella E. Devinsky O. (1999). Vagal nerve stimulator: a new approach to medically refractory epilepsy. N. J. Med. 96, 37–40. PMID: 10384766

[ref11] Floto R. A. Smith K. G. (2003). The vagus nerve, macrophages, and nicotine. Lancet (London, England). 361, 1069–1070. doi: 10.1016/S0140-6736(03)12902-9 12672307

[ref12] Garone C. Donati M. A. Sacchini M. Garcia-Diaz B. Bruno C. Calvo S.. (2013). Mitochondrial encephalomyopathy due to a novel mutation in ACAD9. JAMA Neurol. 70, 1177–1179. doi: 10.1001/jamaneurol.2013.3197, PMID: 23836383 PMC3891824

[ref13] Gofton T. E. Young G. B. (2012). Sepsis-associated encephalopathy. Nat. Rev. Neurol. 8, 557–566. doi: 10.1038/nrneurol.2012.183 22986430

[ref14] Goggins E. Mitani S. Tanaka S. (2022). Clinical perspectives on vagus nerve stimulation: present and future. Clin. Sci. (Lond.) 136, 695–709. doi: 10.1042/CS20210507, PMID: 35536161 PMC9093220

[ref15] He M. Rutledge S. L. Kelly D. R. Palmer C. A. Murdoch G. Majumder N.. (2007). A new genetic disorder in mitochondrial fatty acid beta-oxidation: ACAD9 deficiency. Am. J. Hum. Genet. 81, 87–103. doi: 10.1086/519219, PMID: 17564966 PMC1950923

[ref16] Hotchkiss R. S. Moldawer L. L. Opal S. M. Reinhart K. Turnbull I. R. Vincent J.-L. (2016). Sepsis and septic shock. Nat. Rev. Dis. Primers. 2, 16045–16066. doi: 10.1038/nrdp.2016.45, PMID: 28117397 PMC5538252

[ref17] Iacobone E. Bailly-Salin J. Polito A. Friedman D. Stevens R. D. Sharshar T. (2009). Sepsis-associated encephalopathy and its differential diagnosis. Crit. Care Med. 37, S331–S336. doi: 10.1097/CCM.0b013e3181b6ed58 20046118

[ref18] Imamura Y. Wang H. Matsumoto N. Muroya T. Shimazaki J. Ogura H.. (2011). Interleukin-1beta causes long-term potentiation deficiency in a mouse model of septic encephalopathy. Neuroscience 187, 63–69. doi: 10.1016/j.neuroscience.2011.04.063, PMID: 21571042

[ref19] Imamura Y. Yoshikawa N. Murkami Y. Mitani S. Matsumoto N. Matsumoto H.. (2016). Effect of histone acetylation on N-methyl-D-aspartate 2B receptor subunits and Interleukin-1 receptors in association with nociception-related somatosensory cortex dysfunction in a mouse model of Sepsis. Shock (Augusta, Ga). 45, 660–667. doi: 10.1097/SHK.0000000000000547, PMID: 26682951

[ref20] Joo J. Y. Schaukowitch K. Farbiak L. Kilaru G. Kim T. K. (2016). Stimulus-specific combinatorial functionality of neuronal c-fos enhancers. Nat. Neurosci. 19, 75–83. doi: 10.1038/nn.4170, PMID: 26595656 PMC4696896

[ref21] Jouven X. Empana J. P. Schwartz P. J. Desnos M. Courbon D. Ducimetière P. (2005). Heart-rate profile during exercise as a predictor of sudden death. N. Engl. J. Med. 352, 1951–1958. doi: 10.1056/NEJMoa043012, PMID: 15888695

[ref22] Legon W. Bansal P. Tyshynsky R. Ai L. Mueller J. K. (2018). Transcranial focused ultrasound neuromodulation of the human primary motor cortex. Sci. Rep. 8:10007. doi: 10.1038/s41598-018-28320-1, PMID: 29968768 PMC6030101

[ref23] Li X. Ding Y. Ma Y. Liu Y. Wang Q. Song J.. (2015). Very long-chain acyl-coenzyme a dehydrogenase deficiency in Chinese patients: eight case reports, including one case of prenatal diagnosis. Eur. J. Med. Genet. 58, 134–139. doi: 10.1016/j.ejmg.2015.01.005, PMID: 25652019

[ref24] Lima M. N. Barbosa-Silva M. C. Maron-Gutierrez T. (2022). New perspectives for mesenchymal stromal cells as an adjuvant therapy for infectious disease-associated encephalopathies. Neural Regen. Res. 17, 48–52. doi: 10.4103/1673-5374.314292, PMID: 34100426 PMC8451575

[ref25] Magalhães M. Rodrigues F. P. Chopard M. R. Melo V. C. Melhado A. Oliveira I.. (2015). Neuroprotective body hypothermia among newborns with hypoxic ischemic encephalopathy: three-year experience in a tertiary university hospital. A retrospective observational study. São Paulo Med. J. 133, 314–319. doi: 10.1590/1516-3180.2013.7740026, PMID: 25351640 PMC10876352

[ref26] Maldifassi M. C. Martín-Sánchez C. Atienza G. Cedillo J. L. Arnalich F. Bordas A.. (2018). Interaction of the α7-nicotinic subunit with its human-specific duplicated dupα7 isoform in mammalian cells: relevance in human inflammatory responses. J. Biol. Chem. 293, 13874–13888. doi: 10.1074/jbc.RA118.003443, PMID: 30006348 PMC6130954

[ref27] Manuel T. J. Kusunose J. Zhan X. Lv X. Kang E. Yang A.. (2020). Ultrasound neuromodulation depends on pulse repetition frequency and can modulate inhibitory effects of TTX. Sci. Rep. 10:15347. doi: 10.1038/s41598-020-72189-y, PMID: 32948791 PMC7501284

[ref28] Nedeva C. (2021). Inflammation and cell death of the innate and adaptive immune system during Sepsis. Biomol. Ther. 11, 1–15. doi: 10.3390/biom11071011 PMC8301842 34356636

[ref29] Niessen F. Schaffner F. Furlan-Freguia C. Pawlinski R. Bhattacharjee G. Chun J.. (2008). Dendritic cell PAR1-S1P3 signalling couples coagulation and inflammation. Nature 452, 654–658. doi: 10.1038/nature06663, PMID: 18305483

[ref30] Pavlov V. A. Tracey K. J. (2017). Neural regulation of immunity: molecular mechanisms and clinical translation. Nat. Neurosci. 20, 156–166. doi: 10.1038/nn.4477, PMID: 28092663

[ref31] Peña G. Cai B. Ramos L. Vida G. Deitch E. A. Ulloa L. (2011). Cholinergic regulatory lymphocytes re-establish neuromodulation of innate immune responses in Sepsis. J. Immunol. 187, 718–725. doi: 10.4049/jimmunol.1100013, PMID: 21666060 PMC3131488

[ref32] Pérez-Fraile M. I. González-Elipe J. (1986). Encephalopathy in septic shock with disseminated intravascular coagulation. Rev. Clin. Esp. 178, 202–203. PMID: 3715127

[ref33] Purandare C. S. Dhingra S. Rios R. Vuong C. To T. Hachisuka A.. (2022). Moving bar of light evokes vectorial spatial selectivity in the immobile rat hippocampus. Nature 602, 461–467. doi: 10.1038/s41586-022-04404-x, PMID: 35140401

[ref34] Qian B. F. El-Salhy M. Danielsson A. Shalaby A. Axelsson H. (1999). Effects of unilateral cervical vagotomy on antral endocrine cells in mouse. Histol. Histopathol. 14, 705–709. doi: 10.14670/HH-14.705, PMID: 10425538

[ref35] Qiu Z. Kala S. Guo J. Xian Q. Zhu J. Zhu T.. (2020). Targeted Neurostimulation in mouse brains with non-invasive ultrasound. Cell Rep. 32:108033. doi: 10.1016/j.celrep.2020.108033, PMID: 32814040

[ref36] Rosas-Ballina M. Olofsson P. S. Ochani M. Valdés-Ferrer S. I. Levine Y. A. Reardon C.. (2011). Acetylcholine-synthesizing T cells relay neural signals in a vagus nerve circuit. Science 334, 98–101. doi: 10.1126/science.1209985, PMID: 21921156 PMC4548937

[ref37] Satinoff E. (1978). Neural organization and evolution of thermal regulation in mammals. Science 201, 16–22. doi: 10.1126/science.351802, PMID: 351802

[ref38] Scheffler I. E. (2010). Assembling complex I with ACAD9. Cell Metab. 12, 211–212. doi: 10.1016/j.cmet.2010.08.008, PMID: 20816087

[ref39] Semmler A. Hermann S. Mormann F. Weberpals M. Paxian S. A. Okulla T.. (2008). Sepsis causes neuroinflammation and concomitant decrease of cerebral metabolism. J. Neuroinflammation 5, 38–48. doi: 10.1186/1742-2094-5-38, PMID: 18793399 PMC2553764

[ref40] Shin H. C. Jo B. G. Lee C. Y. Lee K. W. Namgung U. (2019). Hippocampal activation of 5-HT(1B) receptors and BDNF production by vagus nerve stimulation in rats under chronic restraint stress. Eur. J. Neurosci. 50, 1820–1830. doi: 10.1111/ejn.14368, PMID: 30735600

[ref41] Sinsheimer A. Mohsen A. W. Bloom K. Karunanidhi A. Bharathi S. Wu Y. L.. (2021). Development and characterization of a mouse model for Acad9 deficiency. Mol. Genet. Metab. 134, 156–163. doi: 10.1016/j.ymgme.2021.09.002, PMID: 34556413 PMC8588265

[ref42] Sisó S. Jeffrey M. González L. (2010). Sensory circumventricular organs in health and disease. Acta Neuropathol. 120, 689–705. doi: 10.1007/s00401-010-0743-5, PMID: 20830478

[ref43] Song D. Li P. Wang Y. Cao J. (2023). Noninvasive vagus nerve stimulation for migraine: a systematic review and meta-analysis of randomized controlled trials. Front. Neurol. 14, 1–10. doi: 10.3389/fneur.2023.1190062 PMC10213755 37251233

[ref44] Suarez A. N. Hsu T. M. Liu C. M. Noble E. E. Cortella A. M. Nakamoto E. M.. (2018). Gut vagal sensory signaling regulates hippocampus function through multi-order pathways. Nat. Commun. 9:2181. doi: 10.1038/s41467-018-04639-1, PMID: 29872139 PMC5988686

[ref45] Teton Z. E. Blatt D. AlBakry A. Obayashi J. Ozturk G. Hamzaoglu V.. (2019). Natural history of neuromodulation devices and therapies: a patient-centered survival analysis. J. Neurosurg. 132, 1385–1391. doi: 10.3171/2019.2.JNS182450, PMID: 31003217

[ref46] Theodore W. H. Fisher R. S. (2004). Brain stimulation for epilepsy. The Lancet Neurology. 3, 111–118. doi: 10.1016/S1474-4422(03)00664-1 14747003

[ref47] Tracey K. J. (2009). Reflex control of immunity. Nat. Rev. Immunol. 9, 418–428. doi: 10.1038/nri2566, PMID: 19461672 PMC4535331

[ref48] Tufail Y. Yoshihiro A. Pati S. Li M. M. Tyler W. J. (2011). Ultrasonic neuromodulation by brain stimulation with transcranial ultrasound. Nat. Protoc. 6, 1453–1470. doi: 10.1038/nprot.2011.371 21886108

[ref49] van Westerloo D. J. Giebelen I. A. J. Florquin S. Daalhuisen J. Bruno M. J. de Vos A. F.. (2005). The cholinergic anti-inflammatory pathway regulates the host response during septic peritonitis. J. Infect. Dis. 191, 2138–2148. doi: 10.1086/430323, PMID: 15898001

[ref50] VanElzakker M. Fevurly R. D. Breindel T. Spencer R. L. (2008). Environmental novelty is associated with a selective increase in Fos expression in the output elements of the hippocampal formation and the perirhinal cortex. Learn. Mem. 15, 899–908. doi: 10.1101/lm.1196508, PMID: 19050162 PMC2632843

[ref51] Wang H. Imamura Y. Matsumoto N. Yoshikawa N. Nakagawa J. Yamakawa K.. (2013). Matrix Metalloproteinase-9 triggers the gap junction impairment and somatosensory neuronal dysfunction in septic encephalopathy. Biochemistry & Pharmacology: Open Access. 2, 1000108–1000115. doi: 10.4172/2167-0501.1000108

[ref52] Wang H. Liao H. Ochani M. Justiniani M. Lin X. Yang L.. (2004). Cholinergic agonists inhibit HMGB1 release and improve survival in experimental sepsis. Nat. Med. 10, 1216–1221. doi: 10.1038/nm1124, PMID: 15502843

[ref53] Wang H. Yu M. Ochani M. Amella C. A. Tanovic M. Susarla S.. (2003). Nicotinic acetylcholine receptor alpha7 subunit is an essential regulator of inflammation. Nature 421, 384–388. doi: 10.1038/nature01339 12508119

[ref54] Wei D. Y. Goadsby P. J. (2021). Cluster headache pathophysiology – insights from current and emerging treatments. Nat. Rev. Neurol. 17, 308–324. doi: 10.1038/s41582-021-00477-w, PMID: 33782592

[ref55] Wiśniewski J. R. Zougman A. Nagaraj N. Mann M. (2009). Universal sample preparation method for proteome analysis. Nat. Methods 6, 359–362. doi: 10.1038/nmeth.1322 19377485

[ref56] Ye J. Tang S. Meng L. Li X. Wen X. Chen S.. (2018). Ultrasonic control of neural activity through activation of the Mechanosensitive Channel MscL. Nano Lett. 18, 4148–4155. doi: 10.1021/acs.nanolett.8b00935, PMID: 29916253

[ref57] Yu K. Niu X. Krook-Magnuson E. He B. (2021). Intrinsic functional neuron-type selectivity of transcranial focused ultrasound neuromodulation. Nat. Commun. 12:2519. doi: 10.1038/s41467-021-22743-7, PMID: 33947867 PMC8097024

[ref58] Zhang X. Goncalves R. Mosser D. M. (2008). The isolation and characterization of murine macrophages. Curr Protoc Immunol. 1:14. doi: 10.1002/0471142735.im1401s83 PMC2834554 19016445

[ref59] Zhang Q-H Sheng Z-Y Yao Y-M. Septic encephalopathy: when cytokines interact with acetylcholine in the brain. Military medical Research (2014) 1:1–9. doi: 10.1186/2054-9369-1-20 PMC4340341 25722876

