# Supplementary material for: Ultrasound stimulation of the vagal nerve improves acute septic encephalopathy in mice
Source: Front Neurosci. 2023 Jul 17;17:1211608. doi: 10.3389/fnins.2023.1211608 (PMC10388538; doi:10.3389/fnins.2023.1211608)
Supplement: Supplementary file 1 [file Data_Sheet_1.docx]

**Supplemental Information**

**Ultrasound Stimulation of the Vagus Nerve Improves Acute Septic Encephalopathy in Mice**

Yukio Imamura, Hisatake Matsumoto, Jun Imamura, Naoya Matsumoto, Kazuma Yamakawa, Nao Yoshikawa, Yuki Murakami, Satoko Mitani, Junichiro Nakagawa, Tomoki Yamada, Hiroshi Ogura, Jun Oda, Takeshi Shimazu


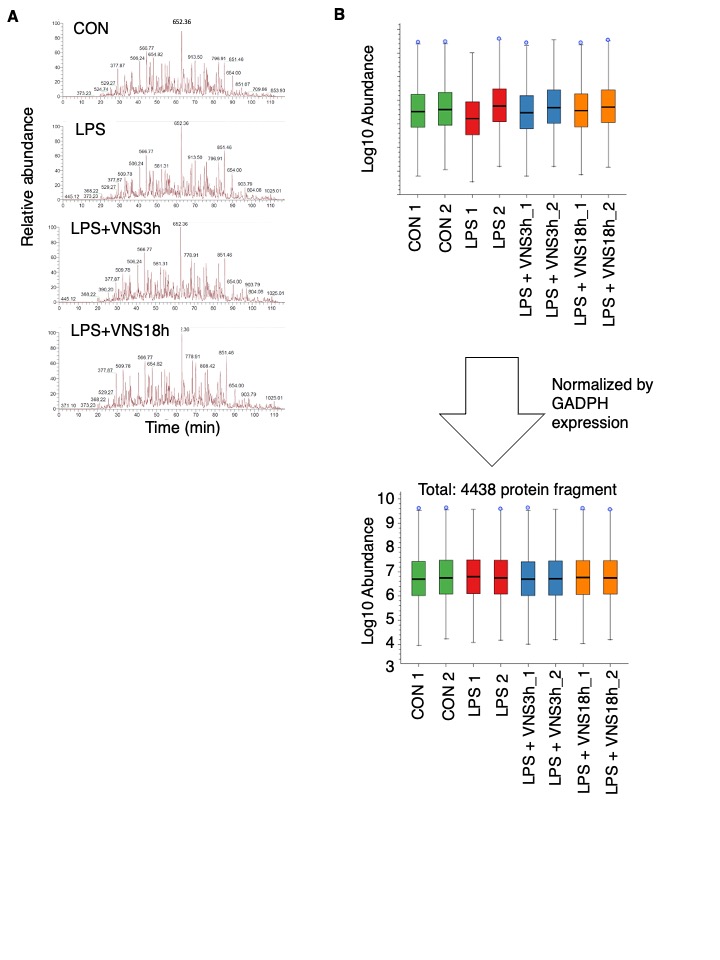


**Supplemental Figure 1 LC-MS/MS**

**(A)**Fragments of proteins from mice groups loaded on LC-MS/MS. **(B)** Scheme for protein fragments normalized by GADPH.


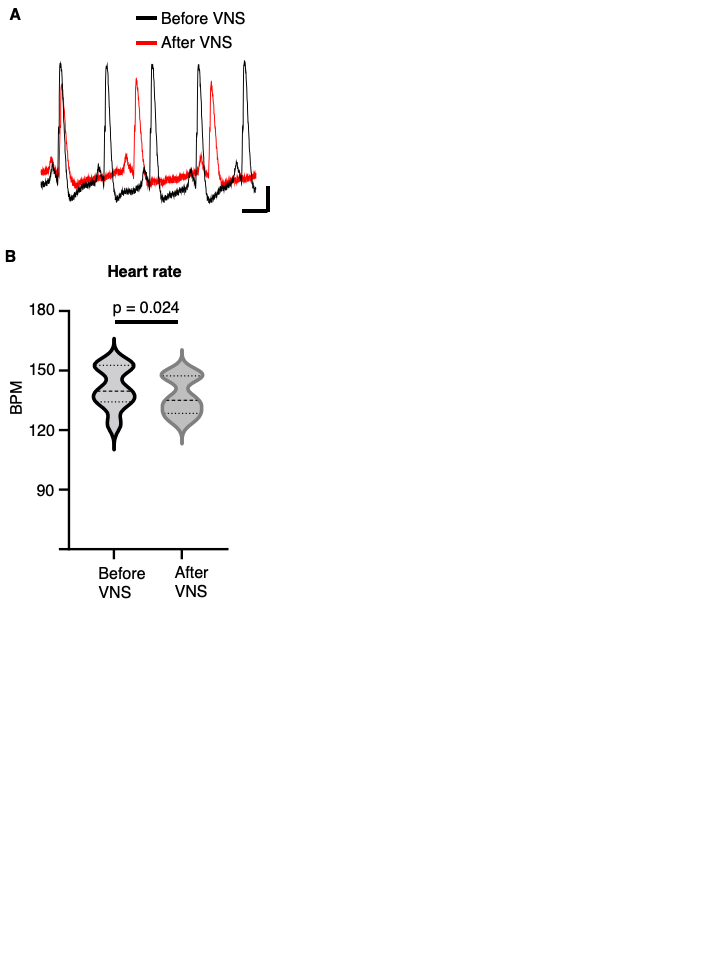


**Supplemental Figure 2 Electrocardiogram before and after the VNS**

(A) ECG. Black: Before VNS, Red: After VNS. (B) Beats per minute. Bar: 0.2mV, 0.2sec. statistical difference was determined by the student t-test. p < 0.05: statistical difference. n = 8 mice in each mouse group.

**Supplemental Figure 3 No significant difference in the temperature at skin after VNS**

We administered VNS to the mice at 1MHz with 50% duty. Statistical difference was determined by one-way ANOVA followed by Tukey’s post-test. p > 0.05 among groups. n = 3 mice for each group.
